# Glycerine in the Arts

**Published:** 1867-12

**Authors:** 


Glycerine in the Arts.—A German chemist, named Pusher, of Nuremberg, reported to the Trades' Union of that place that he had met with great success in using glycerine together with glue. While generally, after the drying of glue, the thing to which it is applied is liable to break, tear, or spring off; if a quantity of glycerine equal to a quarter of the quantity of glue be mixed with it, that defect will entirely disappear. Pusher also makes use of this glue as lining for leather, for making globe frames, and for smoothing parchment and chalk paper. He also uses it for polishing, mixing wax with the glycerine, and using it as an underground for laying on aniline red color. The red was found to excel all others in which the glycerine was not used. The glycerine has also some properties in common with India rubber, for it will blot out pencil marks from paper, so as to leave no mark whatever. A paste made of starch, glycerine, and gypsum, will maintain its plasticity and adhesiveness longer than any other known cement, and does therefore recommend itself for cementing chemical instruments and apparatus used by pharmacists.—*Journal of Applied Chemistry*.

